# Graphene Oxide-Activated Cellular Ceramic Composite Monoliths for Protein Purification

**DOI:** 10.1021/acs.iecr.5c01179

**Published:** 2025-09-08

**Authors:** Eleonora Lalli, Riccardo Onesti, Andrei V. Kovalevsky, Carla A.M. Portugal, João G. Crespo, Giulio C. Sarti, Cristiana Boi

**Affiliations:** † Dipartimento di Ingegneria Civile, Chimica, Ambientale e dei Materiali, DICAM, Alma Mater Studiorum-Università di Bologna, via Terracini 28, Bologna 40131, Italy; ‡ CICECO-Aveiro Institute of Materials, Department of Materials and Ceramic Engineering, 56062 University of Aveiro, Aveiro 3810-193, Portugal; § LAQV-REQUIMTE, Departamento de Química, NOVA School of Science and Technology (NOVA FCT), Universidade Nova de Lisboa, Caparica 2829-516, Portugal; ∥ Instituto de Tecnologia Química e Biológica António Xavier, Universidade NOVA de Lisboa, Av. da República, Oeiras 2780-157, Portugal; ⊥ Department of Chemical and Biomolecular Engineering, North Carolina State University, Raleigh, North Carolina 27695-7905, United States

## Abstract

Cellular Al_2_TiO_5_–Al_2_O_3_ composite ceramic monoliths were prepared and tested as a potential stationary phase for process chromatography. The material was characterized by measuring the pore size distribution and hydraulic permeability. The interstitial porosity, the axial dispersion coefficient, and the height equivalent to a theoretical plate were calculated by pulse tests under nonbinding conditions. The surface of the ceramic material was activated with graphene oxide and then functionalized with Cibacron Blue F3GA. The dynamic binding capacity of the functionalized columns was measured in chromatographic cycles by using bovine serum albumin (BSA) as the target molecule. The experiments showed that the proposed monoliths have a well-defined porous structure, leading to particularly interesting flow properties for chromatographic bioseparations, such as very high permeability and convection, as the main mass transport phenomenon. These results are encouraging for a possible future optimization of the functionalization procedure toward the development of efficient convective media for protein purification.

## Graphical abstract

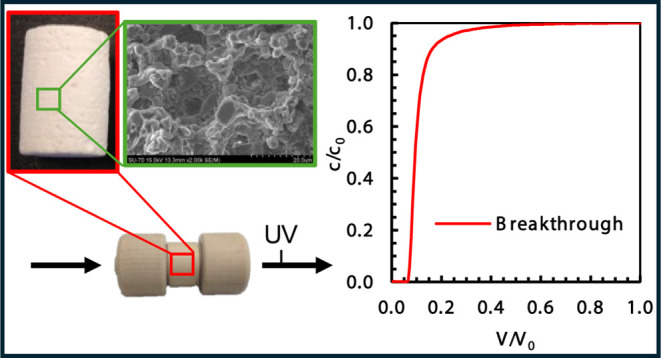

## 1 Introduction

Chromatographic separations based on packed-bed columns represent a fundamental technology for the selective and efficient industrial purification of biomolecules. Columns packed with porous microbeads made of agarose beads, silica particles, glass beads, or various organic polymers with different functionalities are commonly used.^1^ The use of porous beads provides high surface area but also introduces important limitations, most notably slow intraparticle diffusion, which results in flow-dependent binding capacity and long process times.^2^ For analytical applications, this limitation can be mitigated by reducing particle size, while in preparative settings, the associated increase in pressure drop makes significant particle size reduction impractical. The need to find a compromise between these competing factors ultimately imposes a limit on the maximum achievable efficiency and productivity of packed-bed chromatography. In addition, this technology is often associated with high costs of the materials and complex, time-consuming packing procedures and scale-up. For these reasons, there is ongoing research toward the development of novel supports for convective chromatography, such as membranes and monoliths. Compared to packed bed, convective chromatographic materials offer significantly lower surface area. However, this is offset by the elimination of diffusive mass transfer limitations, resulting in a flow-independent binding capacity and resolution and reduced processing time.^2^ Moreover, due to the different stationary phase configuration, monoliths can be prepared and used in several additional formats, such as disks and pipet tips. There is no need for column packing, and the pressure drop per column length is usually lower.^1,3,4^ Monoliths, however, have scale-up challenges of their own, including heat dissipation during polymerization, control over pore size distribution, wall channeling effects, and mechanical integrity when producing large, mechanically stable formats.^5^ In addition, monoliths typically have a significantly lower permeability compared to columns packed with large-diameter particles.^2^

The pore structure and morphological characteristics of monolithic columns make them particularly attractive for the isolation and purification of very large biomolecules, such as plasmids, extracellular vesicles, viruses, and virus-like particles, that cannot enter the small pores of chromatographic beads. With these large molecules, the potential binding capacity of chromatographic particles is significantly reduced since they can only bind to the external bead surface. Monoliths have a more open structure with through-pores, which translates into a more accessible surface area and can provide a greater binding capacity.^6^ As reported by several research groups, monoliths have been successfully used for the isolation and purification of many large biomolecules, such as nucleic acids, plasmid DNA,^7–9^ viruses,^10–12^ virus-like particles,^13,14^ viral vectors,^15^ and extracellular vesicles,^16,17^ demonstrating the superiority of these convective materials with respect to chromatographic beads.

Monoliths are typically characterized by a bimodal pore size distribution, with through-pores in the micrometer range and internal small pores in the nanometer range.^18^ The large proportion of through-pores and their high porosity, usually around 60%, provide monoliths with desirable high permeability.^19,20^

Organic and inorganic monolithic materials may be produced as a continuous porous phase in different shapes and sizes, considering that they both can affect temperature gradients during material preparation, as well as mechanical resistance. Indeed, the flexibility that characterizes the design of these materials makes them suitable for both preparative and analytical purposes,^21^ since the exploitation of convective mass transport can significantly reduce both processing and analytical times.^6,22–24^

The most popular materials for monolith chromatography are represented by organic monoliths, usually made of polyacrylamide and polymethacrylate, which can be produced in different formats,^25–27^ while, among the class of inorganic materials, silica monoliths have often been employed.^25,28^

The present work describes the production and characterization of inorganic cellular ceramic monoliths in view of their application for the chromatographic separation of biomolecules. Ceramic monoliths are highly porous materials whose porosity can be tuned according to the manufacturing technique; their use in filtration and separation requires a flexible and rational design to impart the desired functionalities in terms of the porous structure. The processing method applied for their production takes advantage of recent developments in the preparation of porous cellular ceramics through the emulsification of ceramic suspensions.^29–32^ According to this processing method, a novel and promising porous stationary phase based on cellular Al_2_TiO_5_–Al_2_O_3_ composite ceramics was recently designed and produced by our group to be used as a support matrix for adsorption chromatography.^33^

The cellular Al_2_TiO_5_–Al_2_O_3_ composite monoliths were characterized with respect to their fluid dynamics and transport properties. The porosity, the axial dispersion coefficient, and the height equivalent to a theoretical plate (HETP) were experimentally determined by means of pulse tests and the method of moments.^34^ Pulse tests were performed on columns of different lengths, using several tracers over a wide range of molecular weights under nonbinding conditions. Moreover, the permeability was determined in the flow experiments.

The surface of the ceramic monoliths was functionalized with affinity ligands to enable their chromatographic characterization in terms of binding capacity. Several articles deal with the functionalization of inorganic materials in the form of porous nanoparticles and membranes, using different techniques and reagents. Graphene oxide (GO) represents a relatively new opportunity in terms of surface activation: it is a graphene derivative material characterized by the presence of different functional groups (i.e., hydroxyl, epoxy, and carboxyl groups) and a high surface area (theoretical limit: 2630 m^2^/g). In addition, GO coating can be produced using relatively simple aqueous-phase deposition methods, without the need of high-temperature treatments or complex protocols often required for metallic nanoparticles such as gold or titanium dioxide.^35,36^ Graphene oxide has aroused great interest since it can find applications in many different fields.^37–40^

The functional groups created with GO were used to immobilize an affinity ligand, Cibacron Blue F3GA (CB for simplicity), on the surface. CB belongs to the triazine dye family of affinity ligands, which has found several applications in the field of protein purification.^41–43^ These ligands are able to interact with the active sites of biomolecules by mimicking the structures of coenzymes and enzyme cofactors.^31,44^ Dye ligands are inexpensive and easy to immobilize on surfaces,^45^ which explains the reasons for the success of Cibacron Blue. In fact, this ligand is widely used to capture BSA,^42,45–48^ but also other biomolecules were successfully purified by immobilizing CB on the surfaces of membranes^49–51^ and monoliths.^46,52^

The success of the immobilization was indirectly verified by performing batch and dynamic adsorption tests with pure BSA solutions, and the capacity of the novel ceramic stationary phase was also determined.

The results obtained showed that the design of these novel cellular ceramic monolithic structures was effective in producing materials with very high permeability and promising properties, in terms of fluid dynamics features as well as binding capacity, to be used as alternative chromatographic stationary phases.

## 2 Materials and Methods

The preparation and guidelines to design cellular Al_2_TiO_5_–Al_2_O_3_ monoliths were previously described in detail.^46^ The internal structure of the monoliths was investigated by scanning electron microscopy (SEM) and mercury intrusion porosimetry (MIP) analysis.

Seven columns of similar diameter and different lengths were prepared: three columns were used for fluid dynamic characterization by measuring their permeability and performing pulse tests, and the others were tested in chromatographic cycles to measure the dynamic binding capacity after functionalization with CB.

### 2.1 Chemicals

Bovine serum albumin (BSA), lysozyme, dextran sulfate sodium salt (average molecular weight of 4000 Da), arginine, glycine, acetone, hydrogen peroxide, and buffer chemicals were purchased from Merck S.p.A. (Milan, Italy). Graphene oxide powder was kindly provided by ISOF, CNR (Bologna, Italy). Cibacron Blue F3GA was purchased from Polysciences Europe GmbH (Hirschberg an der Bergstraße, Germany).

### 2.2 Monolith Design and Structure Characterization

The monoliths used in this work were prepared by emulsification of liquid paraffin in aqueous alumina and titania suspensions, using a titania/alumina molar ratio of 0.25 and a liquid paraffin/suspension volume ratio equal to 1. The inherent flexibility of the selected processing procedure actually allows the production of green monoliths with the desired shape, size, and characteristics. For the purpose of this work, the emulsion was cast into a cylindrical shape before the consolidation and drying stages. The samples were fired using a two-step procedure, defined by three parameters: peak temperature, *T*_peak_ = 1550 °C; isothermal treatment temperature, *T*_iso_ = 1300 °C; and time, *t*_iso_ = 4 h. The choice of these sintering conditions is the result of previous intense experimental investigation. Further details on the design, composition, and structural features of the cellular ceramic monoliths can be found elsewhere.^33^ The entire procedure allowed monoliths to be produced with a uniform internal microstructure, suitable for tests and applications, as already demonstrated by the experimental investigation and confirmed by several SEM analyses. Figure 1 shows a representation of the internal structure that can be considered to be representative of the entire monolithic column.

**Figure 1:**
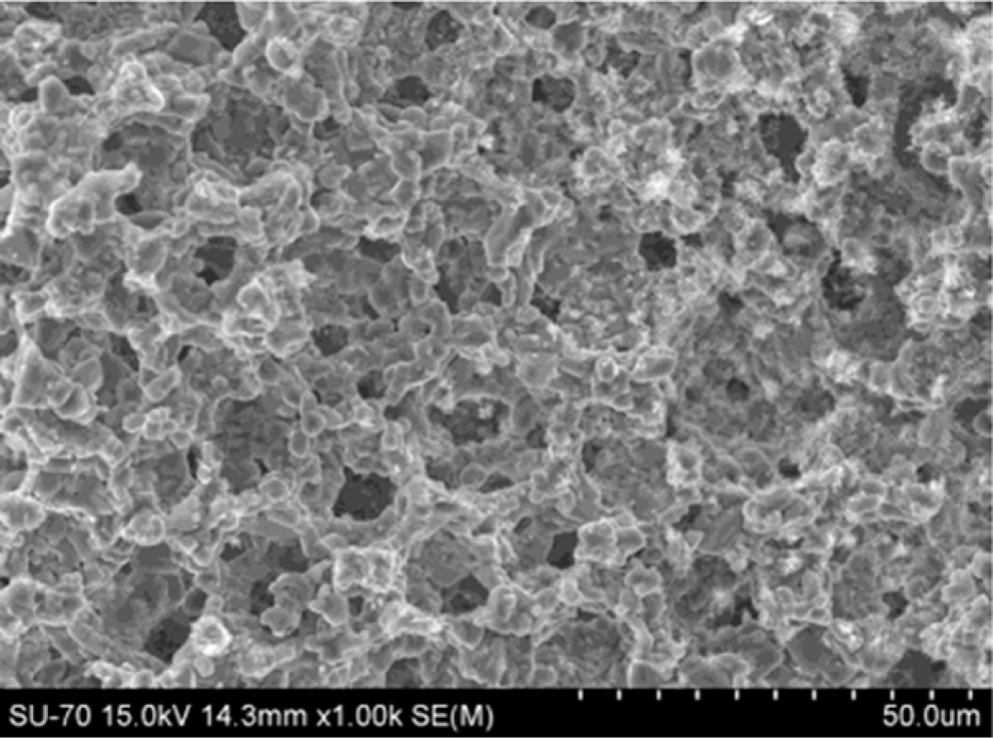
SEM image of the Al_2_O_3_–Al_2_TiO_5_ cellular ceramic monolith microstructure.

#### 2.2.1 Mercury Intrusion Porosimetry

Mercury intrusion porosimetry (MIP) analysis was performed on small pieces of ceramic material to measure the porosity, pore size distribution, and median pore diameter. The measurements were performed using an AutoPore IV 9500 V1.09 instrument (Micromeritics Instrument Corporation, Norcross, USA). The obtained results on porosity were compared to those estimated by moment analysis.

### 2.3 Experimental Setup

Fluid dynamic tests and complete chromatographic cycles were carried out using lab-scale cylindrical ceramic columns of different lengths. The ceramic stationary phase produced exhibited a macroporous layer on its external surface, caused by bubble formation during the sintering process. Therefore, the samples were first polished to remove the outer layer affected by defects and to obtain perfectly cylindrical samples with a diameter of approximately 1.0 cm. The samples were then cut to the desired height and placed inside a rigid plastic ring. Between the ceramic monolith and the rigid plastic ring, thin layers of parafilm and acetic silicone were applied to fill any gaps, to prevent the movement of the monolith inside the holder, and to guarantee the hydraulic seal.

Finally, the columns were placed inside an adjustable-length poly­(ether ether ketone) (PEEK) cell purchased from BIA Separations (Ljubljana, Slovenia) and equipped with two porous disks (frits), one at the column inlet and the other at the column outlet, to ensure a uniform flow distribution of the mobile phase over the entire column cross-sectional area. In Figure 2, a sample of the ceramic column (Figure 2a) and the cell (Figure 2b) are shown.

**Figure 2:**
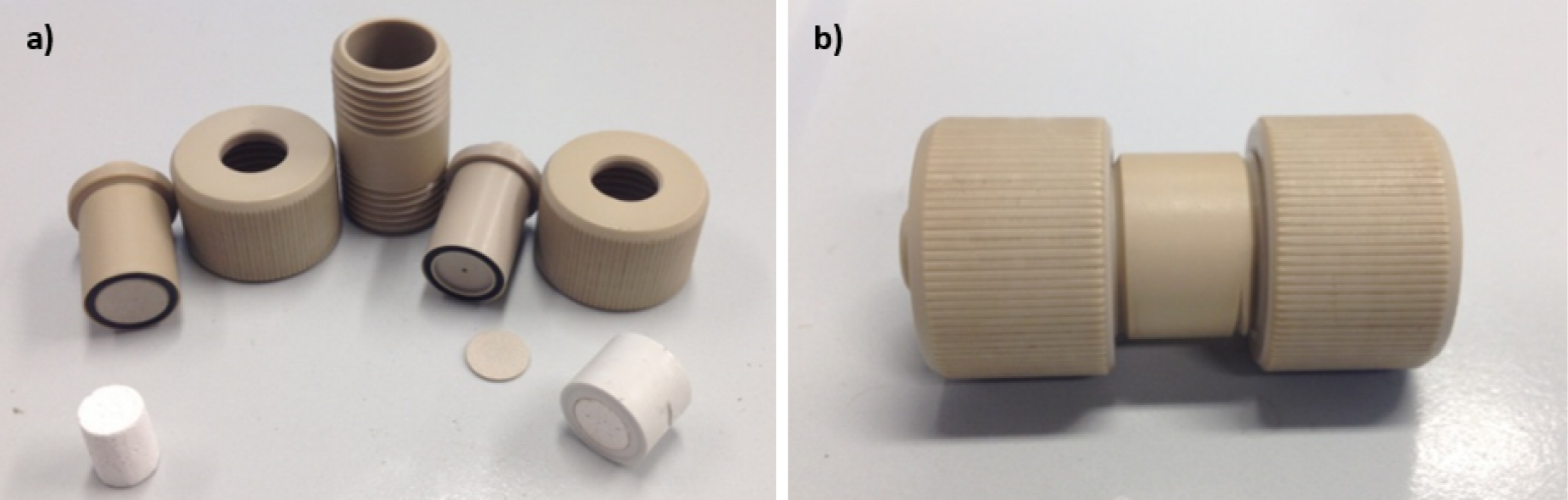
Monolith holder and its component parts: (a) module disassembled with a monolithic column on the left and a monolithic column inside the plastic ring on the right; (b) assembled module.

Three of the seven columns produced were used for the fluid dynamic characterization. Their dimensions are reported in Table 1.

**Table 1:** Dimensions of the Sintered Ceramic Columns Used for Fluid Dynamic Characterization

Sintered columns—fluid dynamic characterization			
	A	B	C
diameter [cm]	1.035	1.170	1.000
height [cm]	0.445	1.174	1.365

Two columns were used for the determination of the dynamic binding capacity. Their dimensions are reported in Table 2.

**Table 2:** Dimensions of the Ceramic Columns Used for Dynamic Adsorption Tests of BSA

	CB columns	
	CB-1	CB-2
diameter [cm]	0.885	0.950
height [cm]	0.470	1.335

The cell was mounted on a Fast Protein Liquid Chromatography (FPLC) apparatus, AKTA Purifier 100 (Cytiva Italy s.r.l., Milan, Italy), which allows the mobile phase flow rate to be adjusted and the main operating parameters, namely, backpressure, conductivity, and solute concentration (by an in-line spectrophotometer), to be continuously monitored immediately downstream of the column.

All buffers were filtered prior to use through a 0.45 μm membrane filter using a vacuum apparatus; protein samples were additionally filtered using a 0.22 μm low-protein-adsorption syringe filter purchased from Sartorius Stedim Biotech GmbH (Göttingen, Germany).

### 2.4 Fluid Dynamic Characterization

#### 2.4.1 Permeability

Water permeability was determined by measuring the pressure drop across the columns when demineralized, filtered water flowed through them at room temperature and at various flow rates ranging from 1 to 20 mL/min, with increments of 5 mL/min. The measured pressure drop was corrected by subtracting the pressure drop observed in the absence of the stationary phase when only the empty column was connected to the FPLC apparatus.

#### 2.4.2 Pulse Tests and Method of Moments

The pulse test consisted of injecting a known amount of an inert tracer and analyzing its distribution in the mobile phase exiting the column. Several tracers were tested to quantify the effect of the molecular weight of the solute on column performance, and their properties are listed in Table 3. The tracers were diluted in a 20 mM phosphate buffer containing 250 mM sodium chloride and 5% (v/v) ethanol to avoid nonspecific interactions, such as electrostatic and hydrophobic interactions. Nonbinding conditions were also ensured by the inertness of the ceramic surface after the high temperatures reached during the firing process.^33^

**Table 3:** Molecular Weight (MW), Diffusivity in Water (*D*_m_), and Concentration (*C*) of the Tracers Used to Perform the Moment Analysis

	MW [g/mol]	*D*_m_ [cm^2^/s]	*C*
Acetone	58.08	8.54 × 10^–6^ ^53^	5% (v/v)
Glycine	75.07	1.10 × 10^–5^ ^54^	400 mM
Arginine	172.20	5.96 × 10^–6^ ^55^	8 mg/mL
Lysozyme	14,400.00	1.11 × 10^–6^ ^56^	4 mg/mL
BSA	66,436.00	6.32 × 10^–7^ ^57^	4 mg/mL

For each tracer and column, different mobile phase velocities (namely 1.0, 2.5, 5.0, and 7.5 mL/min) and injection volumes (namely 10 and 50 μL) were tested to quantify the effects of the amount of solute and the mobile phase velocity on column performance. The tests were carried out on the 3 columns listed in Table 1 and in the absence of the stationary phase, with an empty cell connected to the FPLC, to subtract the effects of all the volumes external to the column. The chromatograms were obtained by measuring UV absorbance (at 210 nm for glycine and arginine, and at 280 nm for all other tracers and were analyzed by the method of moments.^34^ For each condition, the experiments were performed in triplicate, and the average values were considered to reduce the error associated with a single measurement. According to the method of moments, from the analysis of the effluent peaks resulting from small pulse injections of tracers under nonbinding conditions, it is possible to determine the void fraction and the dispersion coefficient that characterize a porous medium.^58^ In fact, the first moment, μ_1_, of an unretained tracer represents the mean residence time of the column and is, therefore, closely related to the structural properties of the porous medium. For a porous monolith, it can be written as

1 $$\mu_{1}=\frac{L}{u_{f}}\varepsilon$$

where *L* is the column height, ε is the void fraction of the column, and *u*_f_ is the superficial velocity.^59^

The axial dispersion coefficient was determined for each tracer starting from the measured values of the second centered moment, $\mu_{2}^{*}$ that for a porous medium is expressed by

2 $$\mu_{2}^{*}=2L\frac{D_{L}}{u_{f}^{3}}\varepsilon^{2}$$

where _L_ is the axial dispersion coefficient. In addition, the first moment and the second centered moment allow to experimentally determine the efficiency of a chromatographic column in terms of height equivalent to a theoretical plate (HETP), according to

3 $$\mathrm{HETP}=\frac{\mu_{2}^{*}}{\mu_{1}^{2}}L$$

By using eq 1 and the relationship between $\mu_{2}^{*}$ and the axial dispersion coefficient [20], eq 3 becomes

4 $$\mathrm{HETP}=\frac{2D_{L}}{u_{f}}$$

### 2.5 Protein Binding Capacity

Several complete chromatographic cycles were performed on columns CB-1 and CB-2. The tests were carried out before and after functionalization with Cibacron Blue F3GA, to obtain the dispersion and breakthrough curves, respectively, and to measure their dynamic binding capacity. To demonstrate the absence of nonspecific adsorption by the unmodified ceramic material, essential for the correct evaluation of the dynamic binding capacity, batch adsorption tests were performed using small fragments of sintered, unmodified samples.

#### 2.5.1 Batch Adsorption for Sintered Unmodified Samples

Batch tests were performed by soaking 0.1188 ± 0.0070 g of fragments of sintered, unmodified samples in 4 mL of 0.5 mg/mL BSA in PBS solution for 24 h, under gentle agitation. The protein concentration was measured at the beginning and end of the adsorption step by measuring the absorbance at 280 nm with a spectrophotometer (UV-1601, Shimadzu Italia, Milan, Italy).

#### 2.5.2 Surface Functionalization

The surface of the monoliths was cleaned with hydrogen peroxide before functionalization to remove any organic contaminants. The monolith samples were immersed in 30% v/v hydrogen peroxide under gentle agitation and kept under vacuum at 80 °C for 4 h. After cleaning, the samples were washed several times with demineralized water and dried overnight at 70 °C.

Due to the inertness of the ceramic samples, a preliminary activation step was performed using an aqueous GO solution that was prepared by dissolving 1 mg of GO powder per mL of demineralized water. The GO exfoliation and delamination into nanosheets were promoted by ultrasonication in a water bath for 2 h.^60,61^ The GO solution was then recirculated through the monoliths for 3 h at room temperature. The monoliths were then subjected to an overnight thermal treatment at 80 °C.

After surface activation, the functionalization using Cibacron Blue F3GA was carried out by recirculating through the columns a 10 mg/mL CB in a 1 M NaOH solution at 80 °C for 3 h. Finally, the samples were thoroughly washed with water and dried at room temperature.

#### 2.5.3 Protein Binding

Protein binding experiments were performed by feeding pure BSA solutions at concentrations ranging from 0.5 to 5 mg/mL, prepared by diluting the protein in 25 mM phosphate buffer containing 0.05 M NaCl, pH 7.4. The same phosphate buffer was also used in the equilibration and washing steps, while a 0.05 M Tris-HCl solution containing 0.05 M NaCl and 0.5 M NaSCN (pH 8.0) was used as an elution buffer. This elution buffer was recommended by the ligand manufacturer and has been previously used with the same ligand in other studies.^62,63^ Protein concentration was measured by absorbance readings at 280 nm by using the FPLC in-line spectrophotometer.

The column performance results reported in Section 3.2 showed that the HETP of the monoliths studied did not change with the mobile phase velocity, while it was significantly affected by column length. Therefore, the effect of the mobile phase velocity was not investigated here, and the dynamic binding capacity was measured by feeding BSA solutions only at 1 mL/min to columns of different lengths, as reported in Table 2. Experiments were performed before and after functionalization to obtain the dispersion curves and to calculate the dynamic binding capacity, respectively. The CB columns were regenerated with 20 mL of 0.5 M NaOH after every two complete chromatographic cycles to restore the binding capacity of the newly modified material. The reproducibility of the dispersion curves was checked by performing duplicate experiments.

## 3 Results and Discussion

### 3.1 Permeability

Considering the values of the flow rate used to carry out the experiments, the water density equal to 1000 kg/m^3^, the water viscosity equal to 0.001 Pa·s, the size of the monoliths, as well as their average pore diameter, it can be seen that the viscous flow conditions are met in all the measurements and, therefore, the permeability can be calculated by applying Darcy’s law:^64^

5 $$\Delta P=\frac{\mu L}{k}u_{f}$$

where Δ*P* is the pressure drop across the column of length *L*, μ is the viscosity of the fluid, and *k* is the superficial velocity-based permeability of the medium.

Figure 3 shows the results obtained for all samples. As expected, the pressure decrease increases linearly with the superficial velocity. The resulting average permeability, *k* = (3.71 ± 2.24) × 10^–13^ m^2^, of the ceramic monoliths is 2 orders of magnitude higher than that of commercial polymeric monoliths (CIM discs; *k* = 5.74 × 10^–15^ m^2^; ε = 0.60,^20^) and more than 2 times higher than that of the MabSelect PrismA packed column (*k* ≈ 1.5 × 10^–13^ m^2^, calculated based on the data provided).^65^ Such high permeability values, combined with the absence of diffusive limitations (as demonstrated in Section 3.2), allow for operation at flow rates up to twice as high as those typically used in conventional chromatographic columns, thereby proportionally reducing the overall process time.

**Figure 3:**
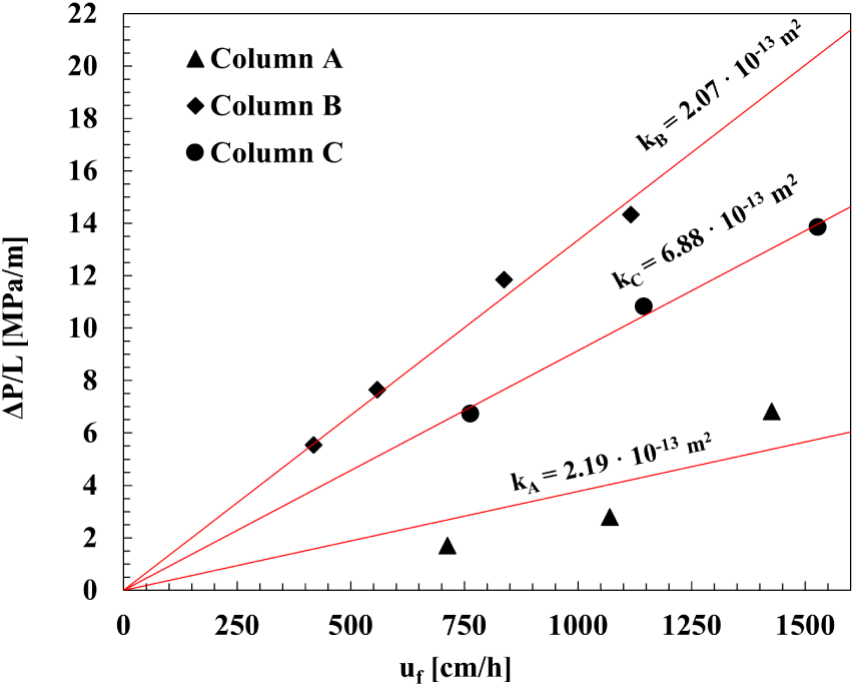
Pressure drops through the monolithic columns against the superficial velocity. Refer to Table 1 for monolith samples dimensions.

### 3.2 Method of Moments

The first moment measured for each tracer molecule at different flow rates was plotted against the theoretically calculated residence time *L*/*u*_f_, and the porosity was obtained from the slope of the straight line of the linear regressions that were, in all cases, characterized by a coefficient of determination *R* ^2^ > 0.98. Contributions from extra-column volumes were taken into account by performing the same analysis on the effluent peaks at the exit of the empty column holder.

Figure 4a shows the results for the measured void fraction as a function of the molecular weight of the tracer molecules. The obtained data were averaged on all experiments performed, and for each tracer, the void fraction is the average of the three samples. The average porosity measured for all of the samples is 57.2%, which confirms the good accessibility of the porous material.

**Figure 4:**
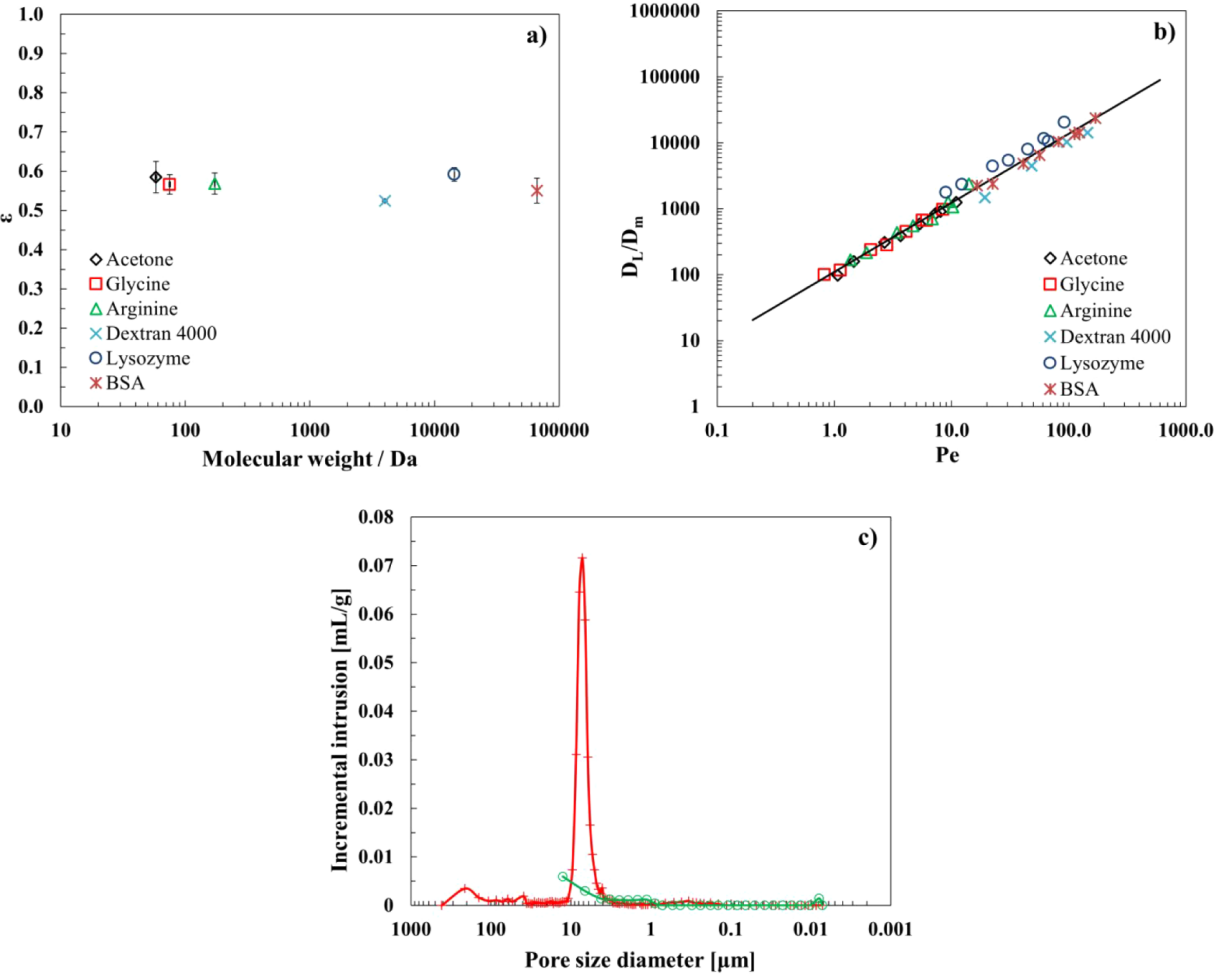
(a) Average void fraction of the ceramic monolithic columns as a function of the tracer molecular weight; (b) axial dispersion coefficient normalized with respect to molecular diffusion against the Péclet number for tracers of different molecular weights; (c) pore size distribution of the ceramic monoliths obtained with the MIP technique during intrusion (red curve) and extrusion (green curve) steps.

The total porosity obtained by mercury intrusion porosimetry (MIP), performed on a very small sample of ceramic material, was 58.5%, which agrees very well with the average porosity measured with the moment analysis for all samples. MIP allowed us to measure the median pore diameter, 6.3 μm, and to obtain information on the pore size distribution, reported in Figure 4c.

Moreover, according to the data presented in Figure 4a, in the range of molecular weights studied, the size of the tracers does not have a strong influence on the measured porosity, contrary to that observed by Herigstad et al. in the case of polymeric monoliths.^20^ In fact, usually a significant decrease in the measured porosity can be observed when using large solutes with a molecular weight in the order of 2 MDa (a value much higher than that of the tracers used in this investigation), and this phenomenon is due to the exclusion of these large molecular weight tracers from a volume fraction containing pores with a radius smaller than the radius of gyration of the tracer.^20,66^

The results obtained from the moment analysis and the pore size distribution measurements by mercury intrusion porosimetry (Figure 4c) allow us to conclude that the pore structure of the ceramic monoliths is very well-defined and that all pores are accessible to the majority of the proteins of interest. This is consistent with the fact that no clear trend of porosity dependence on the molecular weight of the tracers used is observed.

The axial dispersion coefficient was determined for each tracer according to eq 2, where the axial dispersion coefficient was normalized with respect to the molecular diffusion coefficient of each tracer and plotted, on a log–log scale graph, as a function of the reduced velocity, namely the Péclet number (Figure 4b):

6 $$\mathrm{Pe}=\frac{u_{f}d_{p}}{D_{m}}$$

where *D*_m_ is the molecular diffusion coefficient in water and *d*_p_ is the median pore diameter, whose value of 6.3 μm was obtained from MIP analysis.

By fitting the data shown in Figure 4b, the following relation, characterized by a coefficient of determination *R* ^2^ = 0.98, was obtained:

7 $$\frac{D_{L}}{D_{m}}=110.6{\mathrm{Pe}}^{1.0}$$

It was thus demonstrated that the normalized axial dispersion coefficient is directly proportional to the Péclet number or, in view of eq 6, that the axial dispersion coefficient scales linearly with the superficial velocity and is independent of the molecular diffusivity:

8 $$D_{L}\propto u_{f}$$

Regarding the HETP, by combining eqs 4 and 8, it is possible to conclude that for the monolith labeled as column C, it does not depend on the superficial velocity, as shown in Figure 5a. This result confirms what has been previously observed for monoliths and membranes^22,62^ and highlights the potential of these convective media: indeed, the binding capacity of these materials does not depend on the flow rate, and they can provide lower plate heights and higher efficiencies than traditional chromatographic resins, especially when used at high flow rates.^25,67^

**Figure 5:**
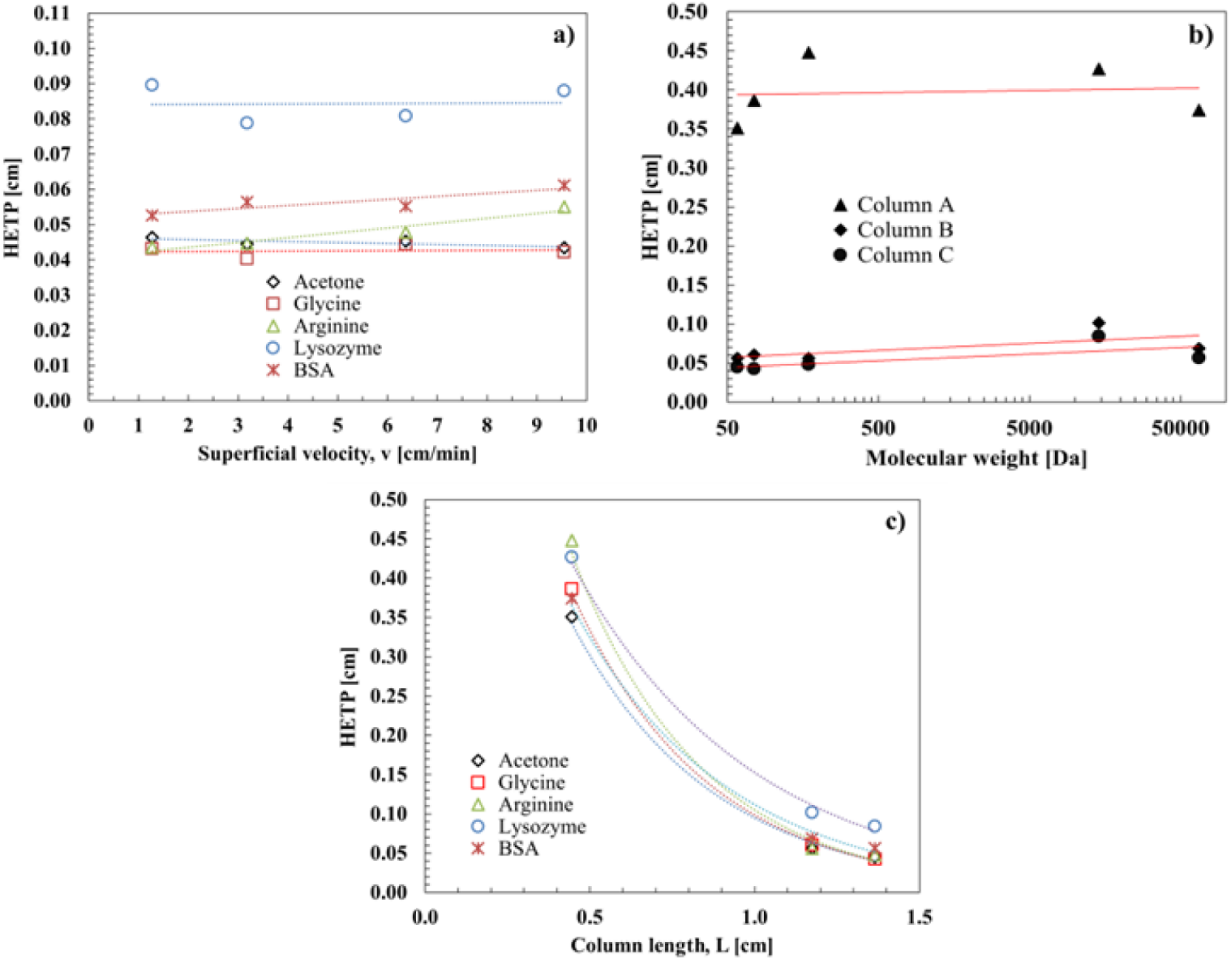
Variation of the HETP with (a) the tracers’ MW and the superficial velocity for monolith C; (b) the tracers’ MW for the three monoliths; and (c) the column length.

In addition, Figure 5a shows that the HETP of monolith C remains constant for the solutes with the smallest molecular weights (namely, acetone, glycine, and arginine), while it is slightly higher for BSA. Notably, the HETP doubles for lysozyme, despite its lower molecular weight compared to BSA, indicating that for this solute the influence of unknown interactions with the stationary phase cannot be excluded. A similar behavior was observed for monolith B, as shown in Figure 5b where the HETP as a function of the tracer molecular weight is reported. Instead, a clear trend cannot be defined for column A. The lack of a clear trend for column A can be explained by analyzing the variation of the HETP with the column length, as shown in Figure 5c.

The HETP decreases significantly with column length because small defects or an uneven distribution of the mobile phase over the cross-sectional area at the column inlet significantly affect the performance of short columns, while their effects are mitigated in longer columns. This result is particularly important because it suggests that better column performance can be achieved by increasing column length.

### 3.3 Protein Binding

For all the batch adsorption experiments performed using sintered unmodified samples, an average adsorption of 0.00 ± 0.00 mg/mg was observed, proving that the ceramic material is completely inert after firing and ensuring that dispersion curves resulting from chromatographic cycles on CB-columns were actually obtained under nonbinding conditions, as well as all moment analysis experiments.

A parameter expressing the performance of chromatographic materials is the dynamic binding capacity at saturation (DBC_100%_), defined by

9 $${\mathrm{DBC}}_{100\%}=\frac{m_{\mathrm{ads},100\%}}{V_{\mathrm{support}}}$$

where *m*_ads,100%_ is the mass of product adsorbed at saturation and *V*_column_ is the volume of the monolithic column.

In Figure 6, an example of the dispersion and the breakthrough curves for samples CB-1 (Figure 6a) and CB-2 (Figure 6b) before and after functionalization obtained by feeding 0.5 mg/mL of a BSA solution is shown.

**Figure 6:**
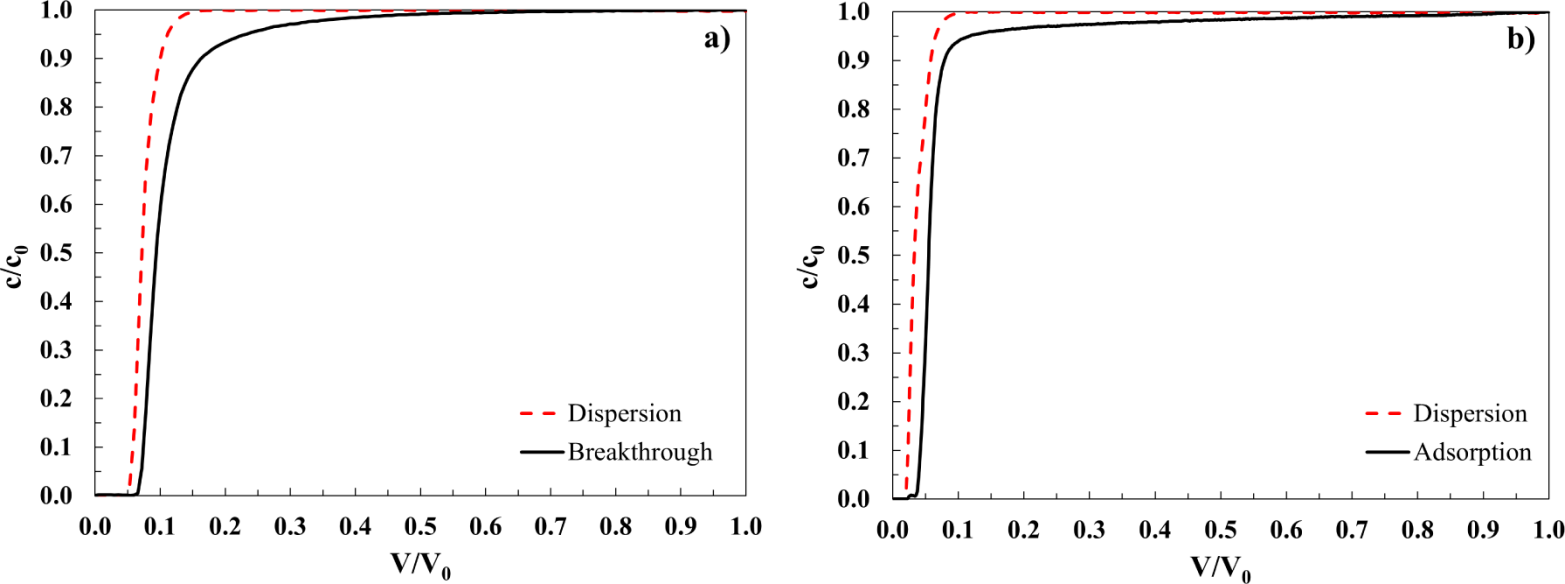
Example of the dispersion and breakthrough curve for: (a) CB-1 column and (b) CB-2 column, at an initial BSA concentration of 0.5 mg/mL.

The dynamic binding capacity was experimentally measured and plotted against the BSA concentration of the feed to obtain the dynamic binding isotherm shown in Figure 7. For each value of BSA concentration in the feed solution, the value of DBC_100%_ is the average of all experiments performed using the two samples. The red line corresponds to the data fitting with the Langmuir model, which gave a maximum binding capacity, *q*_m_, of 4.42 mg/mL, a dissociation constant, *K*_d_, of 2.08 mg/mL with a coefficient of determination *R* ^2^ of 0.92.

**Figure 7:**
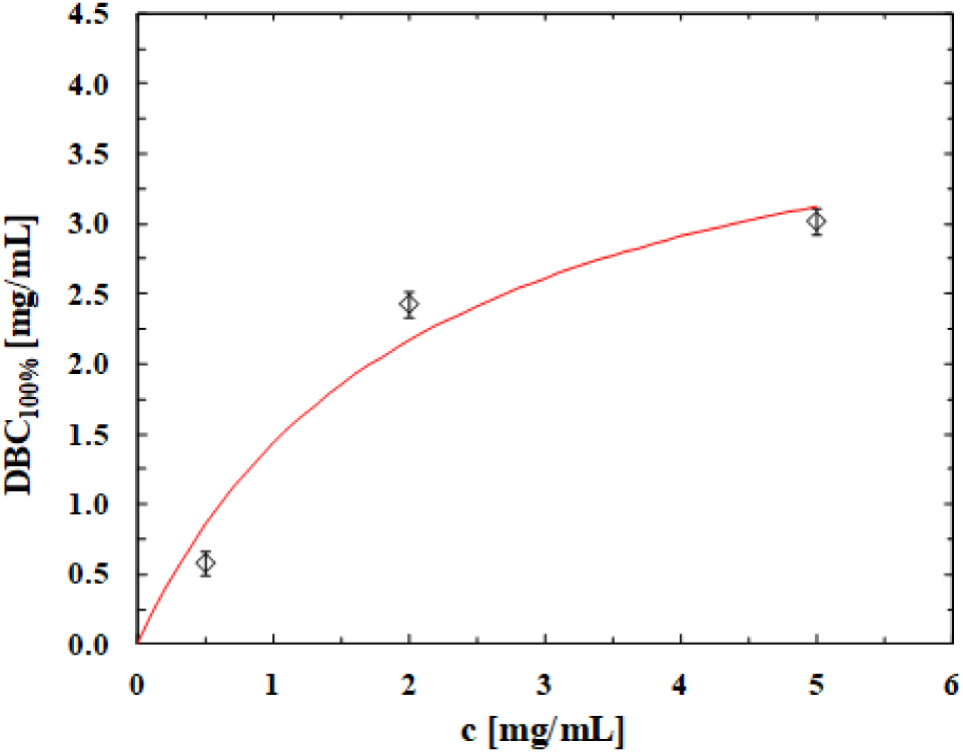
Experimental dynamic adsorption isotherm and relative fitting with the Langmuir model.

These results provide a proof of concept that the newly designed material can be used as a novel chromatographic support for the recovery of biomolecules; however, when compared to commercial polymeric monoliths (CIM discs; *q*_m_ = 7.20 mg/mL; *K*_d_ = 0.11 mg/mL^20^), these results also highlight the need to optimize the functionalization step, for example, by using a more efficient ligand.

## 4 Conclusions

Porous Al_2_TiO_5_–Al_2_O_3_ composite monoliths have been fully characterized in view of their potential use in chromatographic separations as a new porous matrix. The method of moments was applied to obtain the void fraction, the axial dispersion coefficient, and the HETP of the ceramic monoliths, while the systematic measurement of the pressure drops across the porous material allowed us to obtain information on the hydraulic permeability.

The total void fraction of 57.2% is comparable to the literature data for polymeric monoliths and does not show any significant variation as a function of the molecular weight of the tracers used in the method of moments. This is due to the large pore size of the monoliths (with a mean value of 6.3 μm), which allows access to large biomolecules. The method of moments allowed us to calculate the axial dispersion coefficient and to show that the HETP of the material does not depend on the superficial velocity, while it decreases significantly with the column length. These results, combined with a measured permeability of (3.71 ± 2.24)·10^–13^ m^2^, which is 2 orders of magnitude higher compared to that of commercially available polymer monoliths, suggest that the column length can be increased to the benefit of efficiency and surface area.

The measured fluid dynamic properties of this material are very interesting and represent an important starting point for subsequent work: chemical functionalization of the surface and protein binding experiments were performed to prove that the designed materials can indeed be used for chromatographic applications. The obtained value of the maximum binding capacity is 4.42 mg/mL, showing good prospects for future optimization of the functionalization procedure.

## References

[1] Li Z., Rodriguez E., Azaria S., Pekarek A., Hage D. S. (2017). Affinity
Monolith Chromatography: A Review of General Principles and Applications. Electrophoresis.

[2] Martin C., Coyne J., Carta G. (2005). Properties
and Performance of Novel
High-Resolution/High-Permeability Ion-Exchange Media for Protein Chromatography. J. Chromatogr. A.

[3] Vergara-Barberán M., Carrasco-Correa E. J., Lerma-García M.
J., Simó-Alfonso E. F., Herrero-Martínez J. M. (2019). Current Trends in Affinity-Based
Monoliths in Microextraction Approaches: A Review. Anal. Chim. Acta.

[4] Poddar S., Sharmeen S., Hage D. S. (2021). Affinity Monolith
Chromatography:
A Review of General Principles and Recent Developments. Electrophoresis.

[5] Ongkudon C. M., Kansil T., Wong C. (2014). Challenges
and Strategies in the
Preparation of Large-Volume Polymer-Based Monolithic Chromatography
Adsorbents. J. Sep. Sci..

[6] Podgornik A., Krajnc N. L. (2012). Application of Monoliths
for Bioparticle Isolation. J. Sep. Sci..

[7] Kazarian A. A., Barnhart W., Campuzano I. D. G., Cabrera J., Fitch T., Long J., Sham K., Wu B., Murray J. K. (2020). Purification
of Guanine-Quadruplex Using Monolithic Stationary Phase under Ion-Exchange
Conditions. J. Chromatogr. A.

[8] Urthaler J., Schlegl R., Podgornik A., Strancar A., Jungbauer A., Necina R. (2005). Application of Monoliths
for Plasmid DNA Purification
Development and Transfer to Production. J. Chromatogr.
A.

[9] Krajnc N. L., Smrekar F., Cerne J., Raspor P., Modic M., Krgovic D., Strancar A., Podgornik A. (2009). Purification
of Large Plasmids with Methacrylate Monolithic Columns. J. Sep. Sci..

[10] Rogerson T., Xi G., Ampey A., Borman J., Jaroudi S., Pappas D., Linke T. (2023). Purification
of a Recombinant Oncolytic Virus from Clarified Cell
Culture Media by Anion Exchange Monolith Chromatography. Electrophoresis.

[11] Gutiérrez-Aguirre I., Banjac M., Steyer A., Poljŝak-Prijatelj M., Peterka M., Štrancar A., Ravnikar M. (2009). Concentrating Rotaviruses
from Water Samples Using Monolithic Chromatographic Supports. J. Chromatogr. A.

[12] Gerster P., Kopecky E.-M., Hammerschmidt N., Klausberger M., Krammer F., Grabherr R., Mersich C., Urbas L., Kramberger P., Paril T., Schreiner M., Nöbauer K., Razzazi-Fazeli E., Jungbauer A. (2013). Purification
of Infective Baculoviruses by Monoliths. J.
Chromatogr. A.

[13] Pereira
Aguilar P., Reiter K., Wetter V., Steppert P., Maresch D., Ling W. L., Satzer P., Jungbauer A. (2020). Capture and
Purification of Human Immunodeficiency Virus-1 Virus-like Particles:
Convective Media vs Porous Beads. J. Chromatogr.
A.

[14] Burden C. S., Jin J., Podgornik A., Bracewell D. G. (2012). A Monolith Purification Process for
Virus-like Particles from Yeast Homogenate. J. Chromatogr. B.

[15] Bažec K., Kraŝna M., Mihevc A., Leskovec M., Štrancar A., Tajnik Sbaizero M. (2023). Optimization of rAAV Capture Step
Purification Using
SO3Monolith Chromatography. Electrophoresis.

[16] Neumair J., D’Ercole C., De March M., Elsner M., Seidel M., de Marco A. (2023). Macroporous Epoxy-Based Monoliths Functionalized with
Anti-CD63 Nanobodies for Effective Isolation of Extracellular Vesicles
in Urine. Int. J. Mol. Sci..

[17] Davies R. T., Kim J., Jang S. C., Choi E.-J., Gho Y. S., Park J. (2012). Microfluidic
Filtration System to Isolate Extracellular Vesicles from Blood. Lab Chip.

[18] Sproß J., Sinz A. (2011). Monolithic Media for
Applications in Affinity Chromatography. J.
Sep. Sci..

[19] Dimartino S., Herigstad M. O., Boi C., Lalli E., Sarti G. (2016). Experimental
and Theoretical Analysis to Assess the Use of Monolithic Columns in
Process Chromatography. Chem. Eng. Trans..

[20] Herigstad M. O., Dimartino S., Boi C., Sarti G. C. (2015). Experimental Characterization
of the Transport Phenomena, Adsorption, and Elution in a Protein A
Affinity Monolithic Medium. J. Chromatogr. A.

[21] Walch N., Jungbauer A. (2017). Continuous
Desalting of Refolded Protein Solution Improves
Capturing in Ion Exchange Chromatography: A Seamless Process. Biotechnol. J..

[22] Jungbauer A., Hahn R. (2008). Polymethacrylate Monoliths for Preparative and Industrial Separation
of Biomolecular Assemblies. J. Chromatogr. A.

[23] Nascimento A., Rosa S. A. S. L., Mateus M., Azevedo A. M. (2014). Polishing of Monoclonal
Antibodies Streams through Convective Flow Devices. Sep. Purif. Technol..

[24] Tscheliessnig A., Jungbauer A. (2009). High-Performance Monolith Affinity
Chromatography for
Fast Quantitation of Immunoglobulin G. J. Chromatogr.
A.

[25] Mallik R., Hage D. S. (2006). Affinity Monolith Chromatography. J. Sep. Sci..

[26] Tetala K.K., van Beek T.A. (2010). Bioaffinity Chromatography
on Monolithic Supports. J. Sep. Sci.

[27] Svec F., Frechet J. M. J. (1992). Continuous Rods
of Macroporous Polymer as High-Performance
Liquid Chromatography Separation Media. Anal.
Chem..

[28] Vervoort N., Saito H., Nakanishi K., Desmet G. (2005). Experimental Validation
of the Tetrahedral Skeleton Model Pressure Drop Correlation for Silica
Monoliths and the Influence of Column Heterogeneity. Anal. Chem..

[29] Vitorino N., Abrantes J. C. C., Frade J. R. (2013). Cellular
Ceramics Processed by Paraffin
Emulsified Suspensions with Collagen Consolidation. Mater. Lett..

[30] Sanches M. F., Vitorino N., Abrantes J. C. C., Frade J. R., Rodrigues
Neto J. B., Hotza D. (2014). Effects of Processing Parameters
on Cellular Ceramics Obtained by Paraffin Emulsified Suspensions. Ceram. Int..

[31] Vitorino N., Freitas C., Kovalevsky A. V., Abrantes J. C. C., Frade J. R. (2016). Cellular
MgAl2O4 Spinels Prepared by Reactive Sintering of Emulsified Suspensions. Mater. Lett..

[32] Sanches M. F., Vitorino N., Freitas C., Abrantes J. C. C., Frade J. R., Rodrigues Neto J. B., Hotza D. (2015). Cellular Ceramics by
Gelatin Gelcasting
of Emulsified Suspensions with Sunflower Oil. J. Eur. Ceram. Soc..

[33] Lalli E., Vitorino N. M. D., Portugal C. A. M., Crespo J. G., Boi C., Frade J. R., Kovalevsky A. V. (2017). Flexible
Design of Cellular Al2TiO5
and Al2TiO5-Al2O3 Composite Monoliths by Reactive Firing. Mater. Des..

[34] Dimartino S., Boi C., Sarti G. C. (2011). A Validated Model
for the Simulation of Protein Purification
through Affinity Membrane Chromatography. J.
Chromatogr. A.

[35] Dreyer D. R., Park S., Bielawski C. W., Ruoff R. S. (2010). The Chemistry of
Graphene Oxide. Chem. Soc. Rev..

[36] Acquah C., Obeng E. M., Agyei D., Ongkudon C. M., Moy C. K. S., Danquah M. K. (2017). Nano-Doped Monolithic
Materials for Molecular Separation. Separations.

[37] Kashyap S., Pratihar S. K., Behera S. K. (2016). Strong
and Ductile Graphene Oxide
Reinforced PVA Nanocomposites. J. Alloys Compd..

[38] Harb S. V., Pulcinelli S. H., Santilli C. V., Knowles K. M., Hammer P. (2016). A Comparative
Study on Graphene Oxide and Carbon Nanotube Reinforcement of PMMA-Siloxane-Silica
Anticorrosive Coatings. ACS Appl. Mater. Interfaces.

[39] Jiao Y., Zhang J., Liu S., Liang Y., Li S., Zhou H., Zhang J. (2018). The Graphene Oxide Ionic Solvent-Free
Nanofluids and Their Battery Performances. Sci.
Adv. Mater..

[40] Yu Z., Di H., Ma Y., Lv L., Pan Y., Zhang C., He Y. (2015). Fabrication of Graphene Oxide–Alumina Hybrids to Reinforce
the Anti-Corrosion Performance of Composite Epoxy Coatings. Appl. Surf. Sci..

[41] Jin G., Zhang L., Yao Q. (2007). Novel Method for Human Serum Albumin
Adsorption/Separation from Aqueous Solutions and Human Plasma with
Cibacron Blue F3GA-Zn­(II) Attached Microporous Affinity Membranous
Capillaries. J. Membr. Sci..

[42] Denizli A., Pişkin E. (2001). Dye-Ligand
Affinity Systems. J. Biochem Biophys. Methods.

[43] Ma Z., Masaya K., Ramakrishna S. (2006). Immobilization of Cibacron Blue F3GA
on Electrospun Polysulphone Ultra-Fine Fiber Surfaces towards Developing
an Affinity Membrane for Albumin Adsorption. J. Membr. Sci..

[44] Gallant S. R., Koppaka V., Zecherle N. (2008). Dye Ligand Chromatography. Methods Mol. Biol..

[45] Andac C. A., Andac M., Denizli A. (2007). Predicting the Binding
Properties
of Cibacron Blue F3GA in Affinity Separation Systems. Int. J. Biol. Macromol..

[46] Uzun L., Yavuz H., Say R., Ersöz A., Denizli A. (2004). Poly­(Ethylene Dimethacrylate-Glycidyl
Methacrylate)
Monolith as a Stationary Phase in Dye-Affinity Chromatography. Ind. Eng. Chem. Res..

[47] Boyer P. M., Hsu J. T. (1990). Adsorption Equilibrium
of Proteins on a Dye-Ligand
Adsorbent. Biotechnol. Technol..

[48] Tuncel A., Denizli A., Purvis D., Lowe C. R., Pişkin E. (1993). Cibacron Blue
F3G-A-Attached Monosize Poly­(Vinyl Alcohol)-Coated Polystyrene Microspheres
for Specific Albumin Adsorption. J. Chromatogr.
A.

[49] Nie H.-L., Zhu L.-M. (2007). Adsorption of Papain with Cibacron
Blue F3GA Carrying
Chitosan-Coated Nylon Affinity Membranes. Int.
J. Biol. Macromol..

[50] Arica M. Y., Denizli A., Salih B., Piskin E., Hasirci V. (1997). Catalase Adsorption
onto Cibacron Blue F3GA and Fe­(III)-Derivatized Poly­(Hydroxyethyl
Methacrylate) Membranes and Application to a Continuous System. J. Membr. Sci..

[51] Champluvier B., Kula M. R. (1992). Dye-Ligand Membranes
as Selective Adsorbents for Rapid
Purification of Enzymes: A Case Study. Biotechnol.
Bioeng..

[52] Demiryas N., Tüzmen N., Galaev I. Y., Pişkin E., Denizli A. (2007). Poly­(Acrylamide-Allyl Glycidyl Ether) Cryogel as a
Novel Stationary Phase in Dye-Affinity Chromatography. J. Appl. Polym. Sci..

[53] Anderson D. K., Hall J. R., Babb A. L. (1958). Mutual
Diffusion in Non-Ideal Binary
Liquid Mixtures. J. Phys. Chem..

[54] Gnanasambandam S., Hu Z., Jiang J., Rajagopalan R. (2009). Force Field for Molecular Dynamics
Studies of Glycine/Water Mixtures in Crystal/Solution Environments. J. Phys. Chem. B.

[55] Germann M. W., Turner T., Allison S. A. (2007). Translational
Diffusion Constants
of the Amino Acids: Measurement by NMR and Their Use in Modeling the
Transport of Peptides. J. Phys. Chem. A.

[56] Kommedal R., Milferstedt K., Bakke R., Morgenroth E. (2006). Effects of
Initial Molecular Weight on Removal Rate of Dextran in Biofilms. Water Res..

[57] Brune D., Kim S. (1993). Predicting Protein
Diffusion Coefficients. Proc. Natl. Acad. Sci.
U. S. A..

[58] Arnold F. H., Blanch H. W., Wilke C. R. (1985). Analysis of Affinity Separations:
I: Predicting the Performance of Affinity Adsorbers. Chem. Eng. J..

[59] Guiochon, G. A. ; Felinger, A. ; Shirazi, D. G. G. Fundamentals of Preparative and Nonlinear Chromatography, 2nd ed.; Elsevier Inc.: San Diego, 2006. 10.1016/j.chroma.2016.03.050 27079748

[60] Shen J., Hu Y., Shi M., Lu X., Qin C., Li C., Ye M. (2009). Fast and Facile Preparation of Graphene Oxide and Reduced Graphene
Oxide Nanoplatelets. Chem. Mater..

[61] Lou Y., Liu G., Liu S., Shen J., Jin W. (2014). A Facile Way to Prepare
Ceramic-Supported Graphene Oxide Composite Membrane via Silane-Graft
Modification. Appl. Surf. Sci..

[62] Lalli E., Silva J. S., Boi C., Sarti G. C. (2020). Affinity Membranes
and Monoliths for Protein Purification. Membranes.

[63] De Sousa Silva, J. Confronto Tra Supporti Cromatografici Di Affinità per Separazione Di Proteine. Ph.D. Thesis; Alma Mater Studiorum - università di Bologna, 2013.

[64] Darcy, H. Les Fontaines Publiques de la Ville de Dijon: Exposition et Application des Principes a Suivre et des Formulesa Employer dans les Questions de Distribution d’Eau, 1856, p. 647.

[65] MabSelect PrismA affinity chromatography; Cytiva Life Science. https://www.cytivalifesciences.com accessed 26–June–2025.

[66] Zöchling A., Hahn R., Ahrer K., Urthaler J., Jungbauer A. (2004). Mass Transfer
Characteristics of Plasmids in Monoliths. J.
Sep. Sci..

[67] Mallik R., Xuan H., Hage D. S. (2007). Development of an Affinity Silica
Monolith Containing Α1-Acid Glycoprotein for Chiral Separations. J. Chromatogr. A.

